# Human Cytomegalovirus miR-UL148D Facilitates Latent Viral Infection by Targeting Host Cell Immediate Early Response Gene 5

**DOI:** 10.1371/journal.ppat.1006007

**Published:** 2016-11-08

**Authors:** Chaoyun Pan, Dihan Zhu, Yan Wang, Limin Li, Donghai Li, Fenyong Liu, Chen-Yu Zhang, Ke Zen

**Affiliations:** 1 Jiangsu Engineering Research Center for MicroRNA Biology and Biotechnology, State Key Laboratory of Pharmaceutical Biotechnology, School of Life Sciences, Nanjing University, Nanjing, Jiangsu, China; 2 School of Public Health, University of California at Berkeley, Berkeley, California, Unites States of America; Tulane Health Sciences Center, UNITED STATES

## Abstract

The mechanisms underlying human cytomegalovirus (HCMV) latency remain incompletely understood. Here, we showed that a HCMV-encoded miRNA, miR-UL148D, robustly accumulates during late stages of experimental latent HCMV infection in host cells and promotes HCMV latency by modulating the immediate early response gene 5 (IER5)-cell division cycle 25B (CDC25B) axis in host cells. miR-UL148D inhibited IER5 expression by directly targeting the three-prime untranslated region(3’UTR) of IER5 mRNA and thus rescued CDC25B expression during the establishment of viral latency. Infection with NR-1ΔmiR-UL148D, a derivative of the HCMV clinical strain NR-1 with a miR-UL148D knockout mutation, resulted in sustained induction of IER5 expression but decreased CDC25B expression in host cells. Mechanistically, we further showed that CDC25B plays an important role in suppressing HCMV IE1 and lytic gene transcription by activating cyclin-dependent kinase 1 (CDK-1). Both gain-of-function and lose-of-function assays demonstrated that miR-UL148D promotes HCMV latency by helping maintain CDC25B activity in host cells. These results provide a novel mechanism through which a HCMV miRNA regulates viral latency.

## Author Summary

Human cytomegalovirus (HCMV) is a herpesvirus that is prevalent around the world. Following primary infection, HCMV can persist for the lifetime of a host by establishing a latent infection. While HCMV infection normally causes no clinical symptoms, reactivation of HCMV from latency can cause deadly disease in immunocompromised individuals. HCMV achieves latent infection in hematopoietic progenitor cells by silencing HCMV immediate early (IE) genes, the activation of which serves as the initial step in HCMV replication. HCMV has developed multiple strategies to control the expression of IE genes for latency and reactivation. In the present study, we reported that microRNAs (miRNAs), a class of ~22-nt non-coding nucleotides that post-transcriptionally regulate gene expression, are involved in modulating HCMV latency and reactivation. In particular, we found that HCMV miR-UL148D accumulated in progenitor cells during the establishment of experimental HCMV latency. Furthermore, we identified cellular immediate early response gene 5 (IER5), a p53 target gene, as a novel target of miR-UL148D. Functionally, miR-UL148D efficiently inhibited the up-regulation of IER5 during latent viral infection, maintaining the activity of CDC25B and CDK1 and thus controlling IE1 transcription. In conclusion, our study provides the first evidence that HCMV miR-UL148D facilitates latent viral infection by modulating the IER5-CDC25B axis in host cells.

## Introduction

Human cytomegalovirus (HCMV), a member of the β-herpesvirus subfamily, is a ubiquitous human virus that has infected up to 90% of the adult population worldwide [1]. Although HCMV infection rarely causes clinically symptomatic disease in immunocompetent healthy hosts, HCMV can establish a latent infection in hosts. Reactivation of HCMV from latency in immunocompromised people, such as AIDS patients, solid organ transplant recipients and neonates, can lead to severe morbidity and mortality [2]. The effects of HCMV-mediated disease in such patients have also highlighted the possible role of the virus in the development of cancer and inflammatory diseases such as vascular diseases and autoimmune diseases [3, 4]. Although previous evidence has suggested that various viral and cellular factors are involved in the establishment of latent HCMV infection [5–10], the mechanisms underlying this type of infection remain incompletely understood.

Latent HCMV infection is initiated by silencing HCMV immediate early (IE) genes. HCMV IE gene products, especially the major IE (MIE) proteins IE1 and IE2, initiate the HCMV lytic cycle by activating the expression of a cascade of early and late viral genes [11, 12]. In latently infected cells, the expression of the MIE gene is blocked, which consequently restricts the expression of most viral genes. Thus, MIE gene silencing is critical for the establishment of viral latency. Although the underlying mechanism remains unclear, recent studies have shown that cellular cyclin-dependent kinase (CDK) is involved in modulating the persistence or latency of HCMV infection. CDK1/2 can directly inhibit IE1 and IE2 expression and facilitate viral latency, and pharmaceutical inhibition of CDK activates IE gene expression and thus precludes HCMV latency and contributes to lytic viral replication [13, 14]. Previous work has demonstrated that HCMV infection elicits cell damage responses and results in the dysregulation of p53 and CDK activity in host cells [15–17]. However, how cellular CDK activity is regulated during latent HCMV infection remains unclear.

MicroRNAs (miRNAs), a class of ~22-nt non-coding nucleotides that post-transcriptionally regulate gene expression, constitute a novel gene regulatory network that plays a critical role in almost all fundamental biological processes [18, 19]. Herpesviruses, including Epstein–Barr virus (EBV), Kaposi’s sarcoma-associated herpesvirus (KSHV), herpes simplex virus 1 (HSV-1) and HCMV, encode the majority of the 250+ reported virally encoded miRNAs [20, 21]. Herpesvirus miRNAs target both viral and cellular genes to modulate various aspects of virus and cell biology, including viral replication [22, 23], cell apoptosis, the cell cycle, host immune responses [24–30] and, most importantly, the establishment and maintenance of viral latency. Various miRNAs encoded by EBV, KSHV and HSV are abundantly expressed during viral latency and may contribute to the establishment or maintenance of this latency by inhibiting viral IE genes or immune surveillance [31–33]. miRNAs may also play a role in HCMV latency. Grey *et al*. [22] and Murphy *et al*. [34] reported that hcmv-miR-UL112-1 inhibits the expression of HCMV IE1, possibly facilitating the establishment and maintenance of viral latency. Moreover, Meshesha *et al*. [35] tested 20 HCMV miRNAs and found eight miRNAs, including miR-UL112-5p and 3p, miR-UL36-5p and 3p, miR-UL22A-5p and -3p, miR-US29-3p and US22-5p, that may promote viral latency in fresh human PBMCs. Recent studies have also demonstrated that HCMV utilizes cellular miRNAs to promote viral latency and to regulate viral reactivation [36, 37].

In the present study, we established an experimental HCMV latency model with the HCMV clinical strain NR-1 in Kasumi-3 cells and CD34^+^ primary hematopoietic progenitor cells (HPCs) and then monitored HCMV-encoded miRNAs at various stages of HCMV infection. We found that miR-UL148D abundantly accumulated during the late stage of HCMV infection, and knockout of miR-UL148D impaired the capacity of HCMV to achieve experimental latency. Mechanistically, we showed that miR-UL148D directly targeted IER5 and maintained the expression level of CDC25B, a molecule that can inhibit viral IE1 expression by activating CDK-1. In contrast, an NR-1 strain with a specific miR-UL148D deletion did not suppress the continuous induction of IER5, leading to low levels of CDC25B and the eventual failure of latent infection in host cells.

## Results

### miR-UL148D accumulated in host cells during experimental HCMV latency

**Fig 1:**
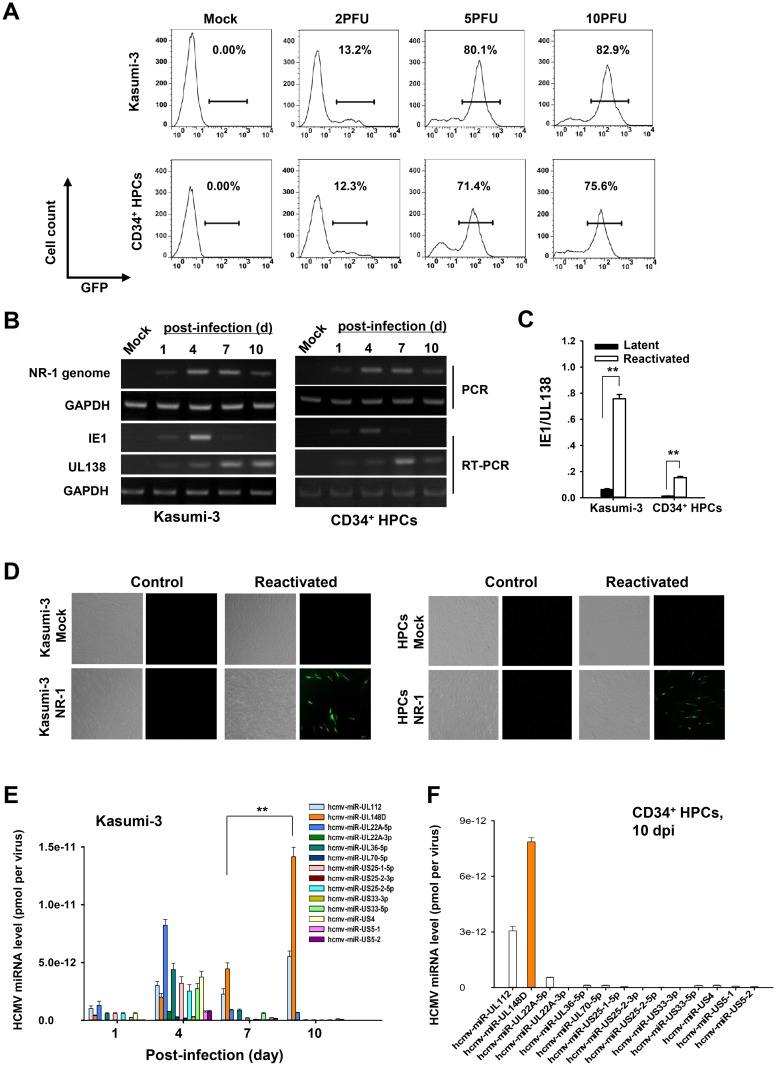
MiR-UL148D robustly accumulates in CD34^+^ progenitor cells during the establishment of experimental HCMV latency. (A) Kasumi-3 and CD34^+^ HPCs were efficiently infected with the NR-1 strain of HCMV. Kasumi-3 and HPCs were either mock-infected or infected with a GFP-expressing NR-1 strain at the indicated multiplicities of infection (MOIs). Two days later, the cells were analyzed for GFP expression by flow cytometry. An MOI of 5 was used for the following experiment. (B) The maintenance of the NR-1 genome, the suppression of viral IE1 and the presence of latency-associated UL138 over a 10-day time course. DNA and total RNA were isolated from Kasumi-3 cells and HPCs at various time points after infection. Viral genomic DNA was assayed by PCR, and RNA molecules encoding IE1 and UL138 were assayed by RT-PCR. In both cases, gel electrophoresis was used to detect the products of the reactions. (C) Reactivation of NR-1 virus in infected Kasumi-3 cells and HPCs. Kasumi-3 cells and HPCs were latently infected along a 10-day time course. Then, a subset of each cell population was cultured for an additional 2 days under conditions favoring lytic reactivation: Kasumi-3 cells were exposed to TPA, while HPCs were grown in reactivation medium. Following this, total RNA was extracted from the cells, and the ratio of IE1 to UL138 cDNA expression was assessed by qRT-PCR in triplicate. (D) Release of infectious progeny virions in latently infected Kasumi-3 cells and HPCs following reactivation treatment. Latently infected or mock-infected Kasumi-3 cells and HPCs were cultured under conditions favoring lytic reactivation (described above) or control conditions for 6 days, after which the cells were washed with PBS and co-cultured with HFFs for 2 days. Then, the Kasumi-3 cells were removed from the co-cultures, and the HFFs were washed with PBS and cultured for an additional 5 days for fluorescence microscopy analysis of GFP-positive plaques. (E) miR-UL148D showed robust accumulation during the establishment of experimental HCMV latency in Kasumi-3 cells. In total, 20,000 infected cells were harvested for the isolation of total RNA and DNA at each indicted time point along the 10-day time course. Viral DNA was first quantified by qPCR, and then absolute viral genomes copies were calculated by generating a standard curve. HCMV miRNAs were then assayed with a HCMV miRNA probe kit, and their levels were calculated using a standard curve. The HCMV miRNA level per virus was calculated by dividing the amount of each HCMV miRNA by the virus copy number. (F) miR-UL148D accumulated in HPCs latently infected with NR-1. HCMV miRNA levels in NR-1-infected HPCs were determined as described above. Values are shown as the mean ± SEM (n = 3). **, P<0.01.

Previous work has demonstrated that HCMV can establish latency in primary hematopoietic cells [6, 9, 38–47] as well as in Kasumi-3 cells [48–50]. Here, we first determined whether the NR-1 clinical strain can achieve latent infection in CD34^+^ hematopoietic progenitor cells (HPCs) and Kasumi-3 cells. To accomplish this, primary CD34^+^ HPCs were isolated from the bone marrow samples of five HCMV-IgG-positive but HCMV-IgM- and HCMV-DNA-negative donors using an anti-CD34 magnetic cell separation kit. Using this method, the percentage of CD34^+^ HPCs in the total isolated cell population reached approximately 90% (S2 Fig). Then, Kasumi-3 cells and the human CD34^+^ HPCs were infected with a GFP-expressing version of the HCMV clinical strain NR-1. As shown in Fig 1A, at 48 hours post-infection with GFP-expressing NR-1 at a multiplicity of 2 PFU/cell, GFP expression was detected in 13.2% of the Kasumi-3 cells and 12.3% of the CD34^+^ HPCs. Interestingly, at a multiplicity of 5 PFU/cell, the percentages of infected Kasumi-3 cells and CD34^+^ HPCs increased significantly to 80.0% and 71.4%, respectively (Fig 1A). Compared to other BAC-derived virus, including Towne and Ad169, NR-1 had a much higher infection efficiency at MOI of 5 (S1 Fig). The infection efficiency of NR-1 did not increase significantly when MOI of 10 was used (Fig 1A). We also observed that Kasumi-3 and CD34^+^ HPCs infected with HCMV virus at MOI of 5 maintained high viability (S1 Fig). Most CD34^+^ HPCs also maintained a progenitor phenotype at 10 days post-infection of NR-1 at a multiplicity of 5 PFU/cell (S2 Fig). Therefore, a multiplicity of 5 PFU/cell was used for all remaining latent infection experiments.

The hallmark of HCMV latency is the maintenance of the viral genome with very limited viral gene transcription. To monitor the status of HCMV transcription in Kasumi-3 cells and CD34^+^ HPCs, the presence of the viral genome (IE1 DNA) and the IE1 transcript (IE1 RNA) was assayed in infected cells at four time points along a 10-day time course. The latency-associated UL138 transcript was also measured as a control. We found that viral genomes increased significantly through 4 days post-infection (dpi) and then reached a plateau, whereas viral IE1 transcripts peaked at 4 dpi and then decreased to undetectable levels by 10 dpi in both Kasumi-3 cells and CD34^+^ HPCs (Fig 1B). As a positive control, the latency-associated UL138 transcript was consistently detected along the 10-day time course (Fig 1B). These data suggest that NR-1 virus was able to enter a quiescent state in both Kasumi-3 cells and CD34^+^ HPCs highlighted by the restriction of viral gene transcription 10 days post-infection. We then induced lytic reactivation in parallel cultures of each cell type following 10 days of latent infection. To accomplish this, infected Kasumi-3 cells were cultured in the presence or absence of 20nM 12-O-tetradecanoylphorbol-13-acetate (TPA), whereas infected primary CD34^+^ HPCs were cultured in reactivation medium for 2 days prior to the harvest of total cellular RNA [12, 50, 51]. Utilizing RT-qPCR, we assessed the expression level of IE1 cDNA relative to that of UL138. Because UL138 was consistently expressed during both lytic and latent HCMV infections, while IE1 was highly expressed during lytic replication but was silenced during viral latency, the ratio of IE1 to UL138 expression can directly reflect the switch from viral latency to reactivation. As shown in Fig 1C, under latent infection, the ratio of IE1/UL138 was low. However, the IE1/UL138 ratio increased significantly upon HCMV reactivation, suggesting that the virus switched from latency to the lytic replication. To further determine if reactivated virus was infectious, we reactivated HCMV in latently infected cells or cells that underwent a mock infection by co-culturing these cells with HFFs for 2 days. Following the co-culture, the HFFs were isolated and monitored for plaque formation after an additional 5 days by fluorescence microscopy. As shown in Fig 1D, HFFs that were co-cultured with re-activated Kasumi-3 cells or CD34^+^ HPCs displayed GFP-positive plaques. In contrast, within the same time frame, no GFP-positive plaques were observed in HFFs that were in contact with infected cells not submitted to reactivation treatment, suggesting that induction of reactivation in latently infected cells promotes the release of infectious progeny virions. Taken together, our results demonstrate that the NR-1 strain can achieve latency in both Kasumi-3 cells and primary CD34^+^ HPCs.

We next analyzed the expressional profiles of 14 HCMV-encoded miRNAs in Kasumi-3 cells along a 10-day time course of infection. To accomplish this, a total of 20,000 infected cells were harvested for the isolation of total RNA and DNA at each indicted time point along the 10-day time course. We first quantified the viral DNA by qPCR and then calculated the absolute viral genome copies according to the generated standard curve. Next, we assayed the HCMV miRNAs with specific HCMV miRNA probe kit and calculated the absolute amount of each HCMV miRNA based on individual miRNA standard curves. The levels of HCMV miRNAs were then normalized to the viral genome copy number. In the latently infected cells, we found that most of the HCMV miRNAs peaked at 4 dpi but decreased to undetectable levels by 10 dpi, although certain HCMV miRNAs, including miR-UL112, miR-UL148D and miR-UL22A-5p, were still readily detectable (Fig 1E). Notably, the level of miR-UL148D gradually increased along the 10-day time course of infection and accumulated to the highest level on 10 dpi (Fig 1E). The presence of high levels of miR-UL148D in host cells during the establishment of HCMV latency was further confirmed in CD34^+^ HPCs. As shown in Fig 1F, when CD34^+^ HPCs were infected with NR-1 at a multiplicity of 5 PFU/cell, miR-UL148D accumulated at high levels by 10 dpi. These results suggest that miR-UL148D robustly accumulates during HCMV latency in both Kasumi-3 cells and primary CD34^+^ HPCs.

### Knockout of miR-UL148D impaired the silencing of IE1 transcription

To explore the potential role of miR-UL148D accumulation in modulating HCMV infection latency, we generated NR-1ΔmiR-UL148D, a mutant NR-1 virus with miR-UL148D knockout. NR-1ΔmiR-UL148D displayed a similar infection efficiency of Kasumi-3 cells and CD34^+^ HPCs (S1 Fig) and viral growth curve (S3A Fig) to NR-1. We also extracted total RNA from HFFs infected with NR-1 or NR-1ΔmiR-UL148D 4 days post-infection and assayed the expression level of miR-UL148D. While readily detectable in the NR-1-infected cells, miR-UL148D was non-detectable in the NR-1ΔmiR-UL148D-infected cells (S3B Fig). These results confirmed the efficiency of the miR-UL148D knockout.

**Fig 2:**
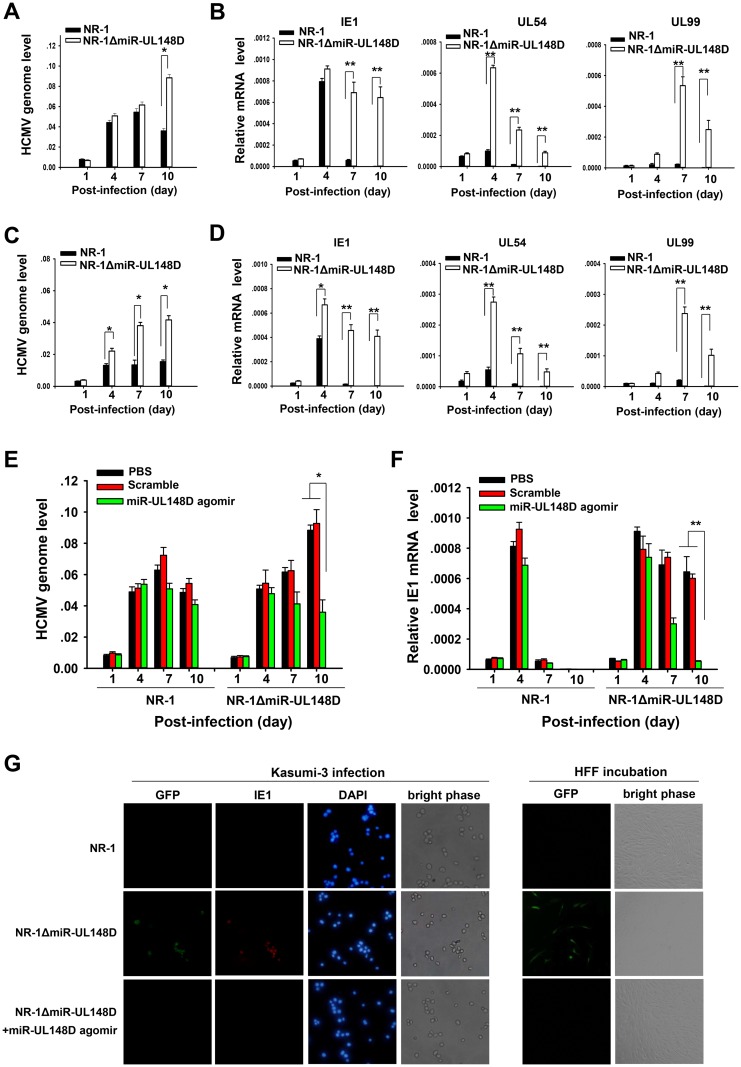
Knockout of miR-UL148D impairs the establishment of experimental NR-1 latency in Kasumi-3 cells. (A, C) HCMV genome copies in Kasumi-3 cells (A) and CD34^+^ HPCs (C) infected with NR-1 or NR-1ΔmiR-UL148D. Total DNA was isolated from the infected cells at various time points after infection, and viral DNA was quantified by qPCR and normalized to cellular GAPDH. (B, D) Representative transcript levels from each class of viral genes in Kasumi-3 cells (B) and HPCs (D) infected with NR-1 or NR-1ΔmiR-UL148D. IE1 (immediately early), UL54 (early lytic transcript), and UL99 (late lytic transcript). Total RNA was isolated from infected cells and assayed by RT-qPCR. Samples were assayed in triplicate, and GAPDH level was used for normalization. (E, F) Restoring miR-UL148D expression via transfection with the miR-UL148D agomir reduced HCMV genome copies (E) and IE1 (F) expression in NR-1ΔmiR-UL148D-infected Kasumi-3 cells. The miR-UL148D agomir was added 24 hours before viral infection, and the culture media was replaced everyday with the addition of fresh agomir. DNA and total RNA were isolated from the Kasumi-3 cells at various time points after infection and quantified by qPCR and RT-qPCR, respectively. Samples were assayed in triplicate, and GAPDH level was used for normalization. Results derived from NR-1-infected Kasumi-3 cells are shown as a control. (G) Both NR-1ΔmiR-UL148D- and NR-1-infected Kasumi-3 cells produced infectious progeny. Infected Kasumi-3 cells harvested 10 days post-infection were stained with a monoclonal antibody against IE1 (clone 1B12, shown in red). GFP (green) and DAPI (blue) served as markers for lytic infection and nuclei, respectively. Infected Kasumi-3 cells were also co-cultured with HFFs. Viral plaque formation in the HFFs (shown by a GFP-positive status) was visualized by fluorescence microscopy. Images were collected using a 40x objective, and representative fields are shown for each infection. Values are shown as the mean ± SEM (n = 3). *, P<0.05. **, P<0.01.

We then utilized qPCR to compare the levels of HCMV genomes and IE1 transcripts in Kasumi-3 cells and CD34^+^ HPCs infected with NR-1 or NR-1ΔmiR-UL148D. Compared to the cells infected with NR-1, the levels of HCMV genomes (Fig 2A and 2C) and IE1 transcripts (Fig 2B and 2D) were significantly higher in the NR-1ΔmiR-UL148D-infected cells on 10 dpi and 7 dpi. These results suggest that miR-UL148D knockout may impair both the silencing of IE1 transcription and viral replication in Kasumi-3 cells and CD34^+^ HPCs. To confirm the above results, we measured the levels of viral early and late gene transcripts (UL54 and UL99, respectively) in the infected cells to monitor the status of viral transcription during the 10 days of infection. We observed that the levels of UL54 and UL99 were significantly higher in the cells infected with NR-1ΔmiR-UL148D than in the cells infected with NR-1, especially on 7 and 10 dpi (Fig 2B and 2D). These results indicated that miR-UL148D knockout possibly induced viral lytic gene transcription. To further validate the role of miR-UL148D in silencing viral IE1, we utilized a chemically modified miR-UL148D mimic, namely, an miR-UL148D agomir, to increase cellular miR-UL148D levels after infection. The agomir was composed of a synthetic RNA duplex that was chemically modified to achieve greater stability and had been previously validated to provide a gain of function *in vivo* [52, 53]. In this experiment, Kasumi-3 cells were incubated with the agomir for 24 h prior to NR-1 or NR-1ΔmiR-UL148D infection. The culture medium was replaced daily, including the addition of fresh agomirs. The viral genome copies and IE1 transcript levels were then measured at four time points along a 10-day time course by qPCR and RT-qPCR, respectively. As shown (Fig 2E and 2F), restoring the expression of miR-UL148D via transfection with the miR-UL148D agomir strongly decreased the HCMV genome copy number and IE1 transcript levels in Kasumi-3 cells infected with NR-1ΔmiR-UL148D.

Consistent with the above findings, immunofluorescence staining results indicated that Kasumi-3 cells infected with NR-1ΔmiR-UL148D were positive for viral GFP and IE1 protein expression 10 days post infection, whereas Kasumi-3 cells infected with NR-1 did not show this phenotype (Fig 2G). Moreover, restoring the expression of miR-UL148D via transfection with the miR-UL148D agomir diminished the expression of IE1 protein and viral GFP in the Kasumi-3 cells infected with NR-1ΔmiR-UL148D (Fig 2G). Importantly, these cells could produce infectious virions that were capable of establishing a lytic infection in HFFs in a co-culture experiment (Fig 2G), suggesting that viruses lacking miR-UL148D favor a lytic rather than a latent infection. Taken together, these data suggest that miR-UL148D plays important roles in silencing the expression of HCMV IE1 and establishing viral latency.

### miR-UL148D helped maintain cellular CDC25B levels by directly targeting IER5

**Fig 3:**
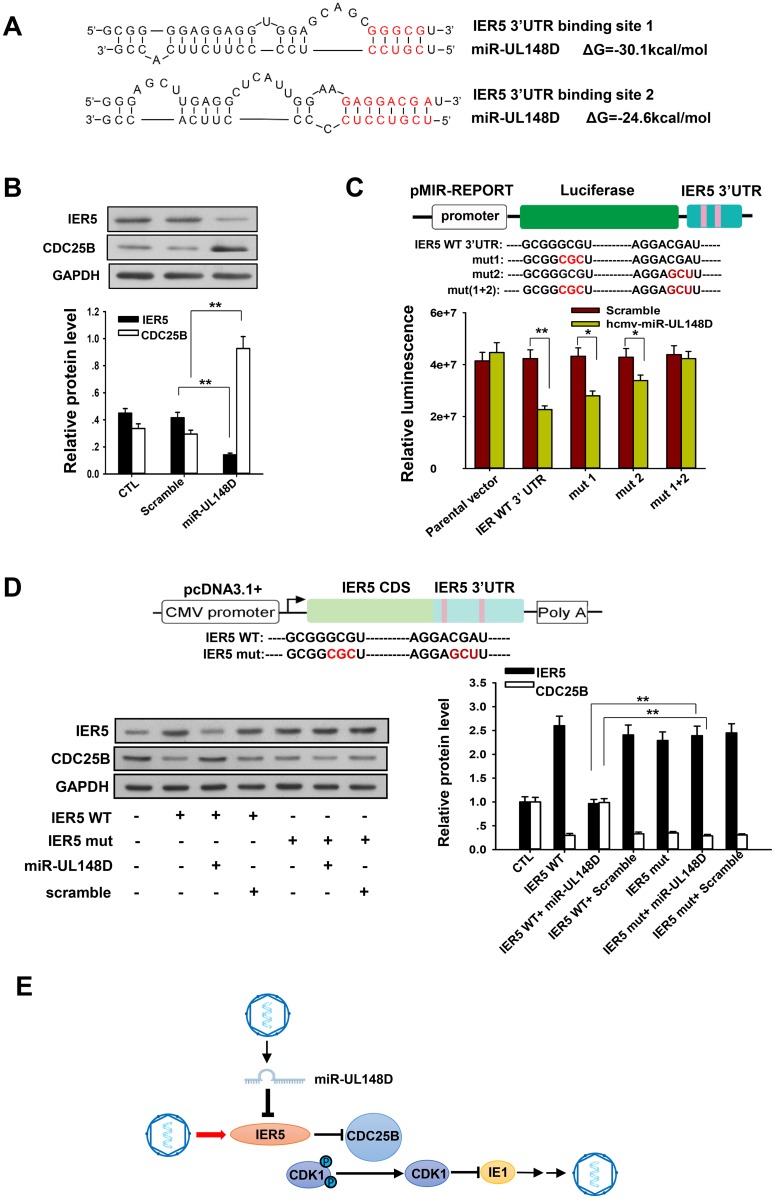
HCMV miR-UL148D directly targets the 3’UTR of IER5 to upregulate CDC25B. (A) Schematic of miR-UL148D-binding sites on IER5, with the two seed-recognizing sites marked in red. The predicted free energy of each hybrid is indicated. (B) Downregulation of IER5 and upregulation of CDC25B protein levels in Kasumi-3 cells induced by miR-UL148D mimics. The protein levels of IER5 and CDC25B in Kasumi-3 cells 48 hours after transfection with miR-UL148D mimics were assayed with western blotting. GAPDH served as an internal control. (C) Upper panel: construction of luciferase reporter that expresses the IER5 3’UTR with or without mutation of miR-UL148D-binding sites. Mutations of the seed-recognizing sites in the IER5 3’UTR are marked in red. Lower panel: firefly luciferase activity in 293T cells that were co-transfected with various luciferase reporter constructs for the IER5 3’UTR and miR-UL148D, normalized to β-gal activity. (D) Upper panel: Construction of an expression vector harboring the ORF and 3’UTR of IER5 with or without mutation of the two miR-UL148D-binding sites. The mutations in the binding sites in the IER5 3’UTR are marked in red. Lower panel: Expression levels of IER5 and CDC25B in Kasumi-3 cells that were co-transfected with wild-type IER5 (IER5 WT) or mutated IER5 (IER5 Mut) and miR-UL148D or a scrambled oligonucleotide. Protein levels were detected by Western blot analysis after a 72-h transfection. The efficiency of transfection was monitored by assaying β-gal activity. (E) Schematic representing the hypothesis for how miR-UL148D facilitates HCMV latency. Values are shown as the mean ± SEM (n = 3). **, P<0.01.

As CDK1/2 could possibly serve as a suppressor of IE1 transcription [14], we first explored whether miR-UL148D can target a CDK1/2-associated protein in host cells to characterize the role of miR-UL148D in silencing IE1. To accomplish this, the computer-aided algorithms TargetScan and RNAhybrid were utilized to identify possible target genes. We identified cellular immediate early response gene 5 (IER5), a slow-kinetic member of the IER gene family, as a potential target gene of miR-UL148D. As shown in Fig 3A, we identified two potential target sites for miR-UL148D located in the proximal region of the IER5 3’UTR (base-pairing between the seed sequence, the first 2–8 bases of miR-UL148D, and the 3’UTR of IER5). Binding site 1 is a non-canonical miRNA-binding site because a loop forms in the binding region. The free energy values of the two binding sites, -30.1 and -24.6 kcal/mol, were well within the range of miRNA-target pairs. Next, we tested whether IER5 serves as a miR-UL148D target. First, we determined the correlation between the expression levels of miR-UL148D, IER5 and CDC25B, a critical CDK-1-regulating phosphatase suppressed by IER5 [54–56], in Kasumi-3 cells. In this experiment, we transfected Kasumi-3 cells with miR-UL148D mimics. Kasumi-3 cells submitted to mock transfection or transfected with scrambled oligonucleotides served as controls. As shown in Fig 3B, the IER5 protein levels in the Kasumi-3 cells transfected with the miR-UL148D mimics were significantly decreased compared with those in the mock-transfected cells or the cells transfected with scrambled oligonucleotides. Concordantly, miR-UL148D transfection resulted in significant upregulation of CDC25B protein expression (Fig 3B).

To determine whether miR-UL148D directly targets the 3’ UTR of IER5, we inserted the entire 3’UTR of IER5, containing two predicted miR-UL148D-binding sites, into a luciferase reporter plasmid (Fig 3C, upper panel). The recombinant reporter plasmid and a β-gal control plasmid were then co-transfected into 293T cells. Either the miR-UL148D mimics or a scramble oligonucleotide were simultaneously introduced into the cells. As shown, transfection of the miR-UL148D mimics significantly inhibited firefly luciferase reporter activity (normalized against β-gal activity) compared to transfection with the scrambled oligonucleotide control (Fig 3C, lower panel). In contrast, co-transfection of 293T cells with the parental luciferase plasmid (without the IER5 3’UTR) and the miR-UL148D mimics did not affect luciferase reporter activity, suggesting that the alteration of luciferase activity caused by miR-UL148D was specific for IER5 (Fig 3C, lower panel). To further validate the binding of miR-UL148D to the two predicted sites on the IER5 3’UTR, we constructed three luciferase reporters in which individual or both miR-UL148D-binding sites were mutated (Fig 3C, upper panel). Luciferase assay results showed that the mutation of individual target sites partially rescued the inhibition caused by miR-UL148D for the WT luciferase construct (10% rescue for site 1 and 25% rescue for site 2) (Fig 3C, lower panel), while the double mutation resulted in a strong rescue (45% rescue compared to the WT construct) (Fig 3C, lower panel), suggesting that the down-regulation of IER caused by miR-UL148D is mediated by the two target sites acting cooperatively. Taken together, these results demonstrate that miR-UL148D directly targets 2 binding site in the 3’UTR of IER5.

Given that IER5 is able to transcriptionally suppress CDC25B [14], we next tested whether miR-UL148D could upregulate CDC25B through direct targeting of IER5 in Kasumi-3 cells. In this experiment, IER5 cDNA (without the 5’UTR) was synthesized chemically and inserted into a pcDNA3.1 plasmid vector (Fig 3D, upper panel). To test the effect of miR-UL148D, a functionally intact IER5-expressing plasmid vector with both miR-UL148D-binding sites mutated (IER5 Mut) was also constructed. Either wild-type IER5 (IER5 WT) or the IER5 Mut plasmid was then transfected into Kasumi-3 cells together with a β-gal plasmid. Either the miR-UL148D mimics or the scrambled oligonucleotide control were simultaneously introduced into the cells. The cells were harvested at 72 h post-transfection for Western blot analysis. As shown, transfection with either the IER5 WT or IER5 Mut plasmid alone increased IER5 levels, resulting in a reduction of CDC25B expression (Fig 3D, lower panel). However, in cells transfected with IER5 WT, transfection with miR-UL148D largely abolished the increase in IER5 level and restored CDC25B expression to the basal level (Fig 3D, lower panel). In contrast, miR-UL148D did not rescue the CDC25B protein expression in cells transfected with the IER5 Mut plasmid (Fig 3D, lower panel). Taken together, these results show that miR-UL148D can upregulate CDC25B by directly targeting the IER5 3’UTR.

As an immediate early response gene, IER5 is constitutively expressed in progenitor cells [57–59] and responds rapidly to external stimulation. Because HCMV infection can lead to the upregulation and activation of p53 [60, 61] and because IER5 is transcribed in a p53-dependent manner [62, 63], HCMV infection may also correspondingly increase the expression of IER5. Moreover, as IER5 can transcriptionally repress CDC25B [56], a key protein involved in activating CDK1 via dephosphorylation of CDK1 at Thr14 and Tyr15 [54–56], we hypothesized that accumulated miR-UL148D modulates IE1 expression by regulating the IER5-CDC25B-CDK1 axis (Fig 3E). According to this model, HCMV infection stimulates IER5 expression, which in turn inhibits CDC25B expression and CDC25B-dependent CDK1 activation, contributing to IE1 transcription and lytic viral replication. Through suppressing IER5 expression, miR-UL148D can serve as a molecular “brake” for this lytic signaling pathway.

### miR-UL148D rescued CDC25B by inhibiting IER5 during experimental HCMV latency

**Fig 4:**
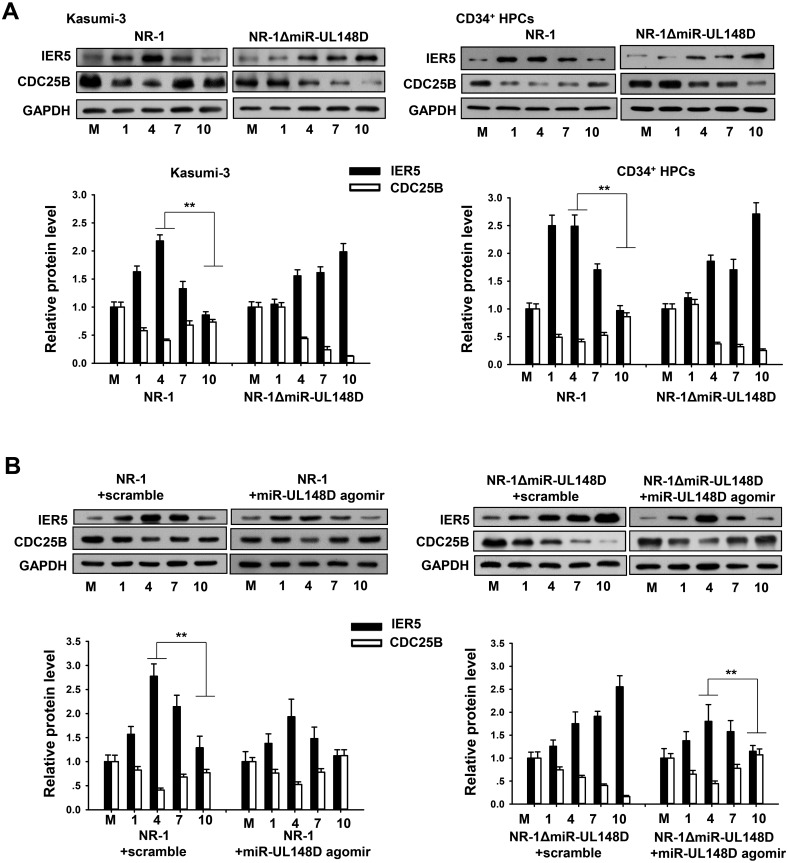
MiR-UL148D reduced IER5 level but enhanced CDC25B expression during the establishment of experimental HCMV latency. (A) Expression levels of IER5 and CDC25B in Kasumi-3 cells and CD34+ HPCs infected with NR-1 or NR-1ΔmiR-UL148D at various time points throughout a 10-day time course. (B) Restoring miR-UL148D expression via transfection with an miR-UL148D agomir suppressed IER5 expression and rescued CDC25B expression in NR-1ΔmiR-UL148D-infected Kasumi-3 cells. The miR-UL148D agomir was added to the cell culture medium 24 hours before viral infection, and the medium was then replaced daily with the inclusion of fresh agomir throughout the 10-day time course. Total cellular protein was isolated from the Kasumi-3 cells at various times after infection and detected by western blotting. The results from Kasumi-3 cells infected with NR-1 virus are shown as a control. Values are shown as the mean ± SEM (n = 3). **, P<0.01.

We next tested whether miR-UL148D affected the expression of IER5 and CDC25B in Kasumi-3 cells and CD34^+^ HPCs authentically infected with HCMV. In this experiment, we infected Kasumi-3 cells and CD34^+^ HPCs with NR-1 or NR-1ΔmiR-UL148D at a multiplicity of 5 PFU/cell. The infected cells were harvested at the indicted time points along a 10-day time course. As shown (Fig 4A), possibly in response to HCMV early infection, the levels of IER5 in the Kasumi-3 cells and CD34^+^ HPCs infected with NR-1 or NR-1ΔmiR-UL148D were increased during the early days (1–4 dpi) of infection. Concordant with this observation, CDC25B levels decreased in Kasumi-3 cells during the early days of infection. However, compared to 4 days post infection, IER5 levels in Kasumi-3 cells and CD34^+^ HPCs infected with NR-1 significantly decreased at later stages of infection (7–10 dpi), while levels of CDC25B expression were restored (Fig 4A). In contrast, possibly due to the lack of miR-UL148D, both the Kasumi-3 cells and the CD34^+^ HPCs that were infected with NR-1ΔmiR-UL148D failed to suppress IER5 expression at later stages of infection, leading to sustained inhibition of CDC25B (Fig 4A). To confirm these results, we also utilized the miR-UL148D agomir to upregulate miR-UL148D levels in Kasumi-3 cells infected with NR-1 or NR-1ΔmiR-UL148D in the same manner as described above. As shown in Fig 4B, in the Kasumi-3 cells infected with NR-1ΔmiR-UL148D, incubation with the miR-UL148D agomir strongly suppressed IER5 expression but restored CDC25B levels at late stages of infection. In the Kasumi-3 cells infected with NR-1, incubation with the miR-UL148D agomir also dampened the robust increase in IER5 expression observed during the early days (1–4 dpi) of infection (Fig 4B). These results collectively suggest that miR-UL148D plays a role in maintaining CDC25B by suppressing HCMV-induced IER5 expression.

### CDC25B expression is essential for silencing HCMV IE1 transcription during experimental HCMV latency

**Fig 5:**
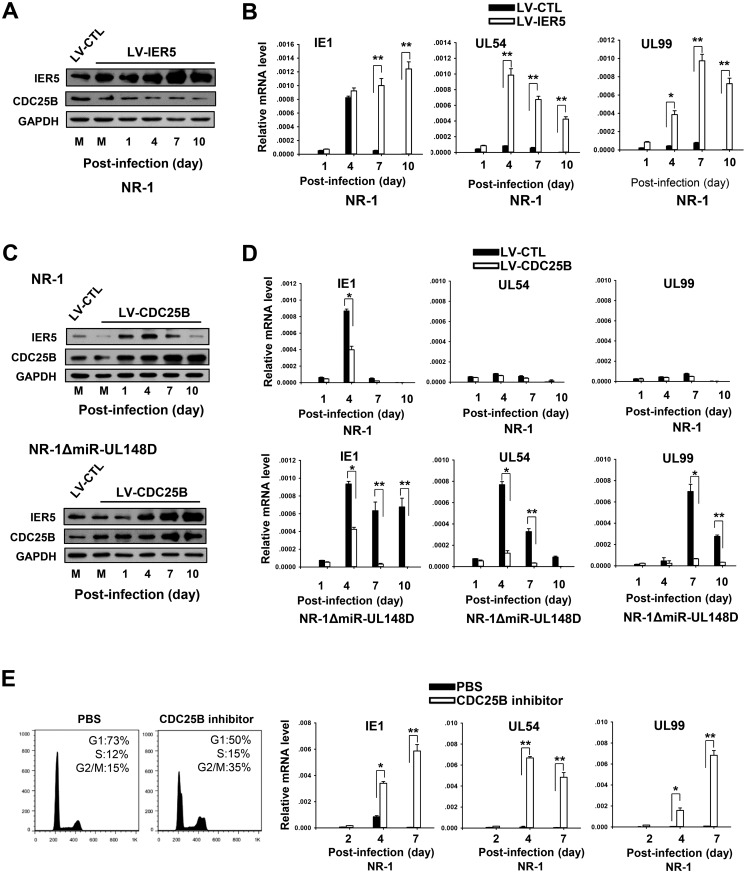
CDC25 expression is essential for silencing IE1 transcription. (A, B) Stable overexpression of IER5 induced viral lytic gene transcription in NR-1-infected Kasumi-3 cells. IER5 was stably overexpressed in Kasumi-3 cells infected with LV-IER5. An empty-backbone lentivirus was used as a control. The cells were then infected with NR-1 virus for a 10-day time course. The infected cells were analyzed for IER5 and CDC25B protein expression (A) and IE1 (immediately early), UL54 (early lytic transcript), and UL99 (late lytic transcript) mRNA expression (B). (C, D) Stable overexpression of CDC25B suppressed viral lytic gene transcription in NR-1ΔmiR-UL148D-infected Kasumi-3 cells. CDC25B was also stably overexpressed in Kasumi-3 cells infected with LV-CDC25B. An empty-backbone lentivirus was used as a control. The cells were then infected with NR-1 or NR-1ΔmiR-UL148D virus for a 10-day time course. IER5 and CDC25B protein expression (C) and IE1, UL54, and UL99 mRNA expression (D) were then measured in the Kasumi-3 cells. (E) Pharmaceutical inhibition of CDC25B efficiently induced viral lytic gene transcription in NR-1-infected cells. After infection with NR-1, Kasumi-3 cells were incubated with NSC663284, a specific CDC25B inhibitor, at a concentration of 5 μM for 48 hours. Then, the treated cells were further cultured in fresh media for 5 days. The DNA content in the treated cells was analyzed 2 dpi. Total RNA was extracted for RT-qPCR analysis of IE1, UL54 and UL99 expression at the indicated time points along a 7-day time course. Values are shown as the mean ± SEM (n = 3). **, P<0.01.

Given that accumulated miR-UL148D could possibly maintain CDC25B expression during experimental HCMV latency, we next tested whether IER5 and CDC25B affected IE1 expression and thus viral latency. In this experiment, we first stably overexpressed IER5 in Kasumi-3 cells using lentivirus (LV-IER5). The lentivirus infection efficiency was approximately 90% at an MOI of 5 (S4 Fig). Forty-eight hours after the lentivirus infection, the Kasumi-3 cells were infected with NR-1. The transcript levels of IE1, UL54 and UL99 were then determined at the indicated time points along a 10-day time course. As shown, infection with LV-IER5 resulted in continuous high-level expression of IER5 in the NR-1-infected Kasumi-3 cells, even at late stages of infection, whereas CDC25B expression was significantly decreased along the 10-day time course (Fig 5A). Importantly, we observed that IER5 overexpression significantly increased the transcript levels of IE1, UL54 and UL99 along the 10-day time course, indicating that IER5 overexpression or inhibition of CDC25B possibly induces viral lytic gene transcription (Fig 5B).

To confirm the role of CDC25B in silencing IE1 transcription, we also overexpressed CDC25B in Kasumi-3 cells using lentivirus vector (LV-CDC25B) (Fig 5C) and measured the transcript levels of IE1, UL54 and UL99 in Kasumi-3 cells infected with NR-1 or NR-1ΔmiR-UL148D at four time points along a 10-day time course. In the Kasumi-3 cells infected with NR-1, the transcripts level of IE1, UL54 and UL99 remained low even at late stages of infection, regardless of the overexpression of CDC25B, although overexpression of CDC25B reduced the increase in IE1 transcription at early stages of infection (Fig 5D). However, in the Kasumi-3 cells infected with NR-1ΔmiR-UL148D, overexpression of CDC25B significantly decreased the transcription of IE1, UL54 and UL99 along the 10-day time course (Fig 5D), suggesting that CDC25B functions as a suppressor of viral IE1 transcription.

We next determined whether direct inhibition of CDC25B impaired the capacity of host cells to silence viral IE1 gene expression. In this experiment, NSC663284, a specific CDC25B inhibitor, was added to the culture medium of NR-1-infected Kasumi-3 cells to a final concentration of 5 μM, and the cells were then incubated for an additional 48 hours. As shown, treating the infected Kasumi-3 cells with the CDC25B inhibitor increased the percentage of cells in G2/M phase from 15% to 35% after 48 hours of incubation (Fig 5E). In the NR-1-infected Kasumi-3 cells, inhibition of CDC25B with NSC663284 resulted in a rapid increase of the transcription of the viral IE1, UL54 and UL99 genes, indicating that direct inhibition of CDC25B strongly promoted the transcription of viral lytic genes. The above results collectively suggest that CDC25B expression plays a critical role in silencing viral IE1 and lytic gene transcription.

### miR-UL148D silences IE1 by promoting CDC25B-induced activation CDK1

**Fig 6:**
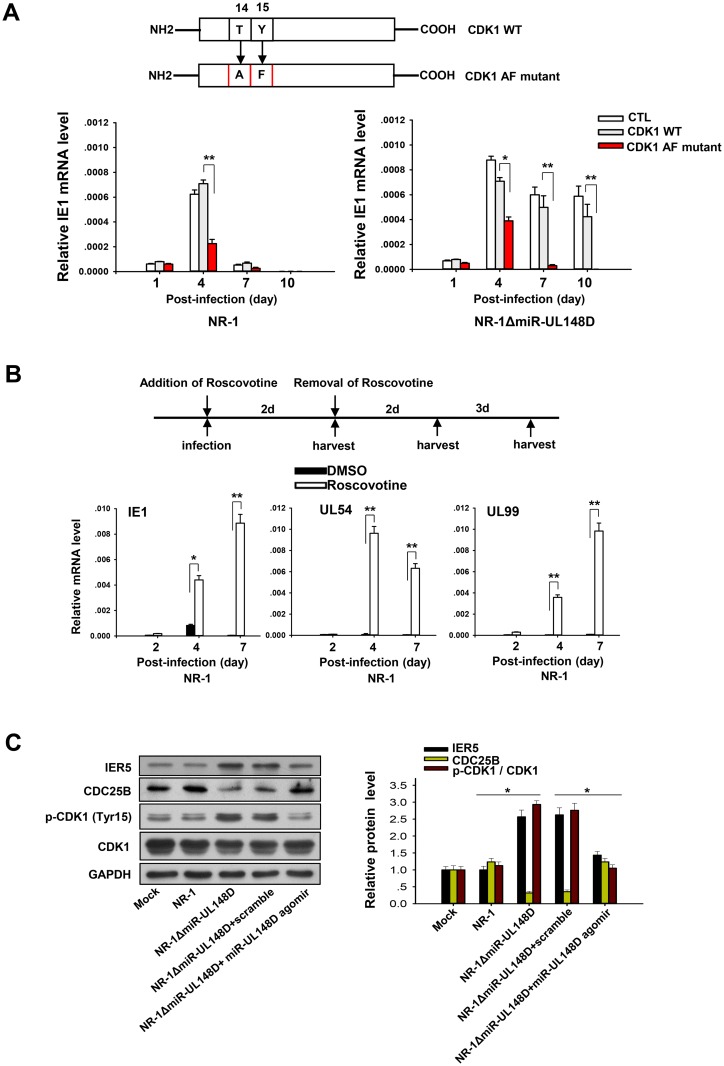
miR-UL148D silences IE1 by activating CDK1. (A) Overexpression of constitutively activated CKD1 efficiently inhibited IE1 transcription. Upper panel: construction of CDK1 WT and CDK1 Mut lentiviruses. CDK1 Mut is constitutively activated through the mutation of two phosphorylation sites. Kasumi-3 cells were infected with lentivirus expressing CDK1 WT or CDK1 Mut 48 h before being subjected to NR-1ΔmiR-UL148D or NR-1 infection over a 10-day time course. An empty-backbone lentivirus was used as a control. The infected cells were harvested at the indicated time points to extract total RNA for RT-qPCR analysis of IE1. (B) CDK1 inhibition induced viral lytic gene transcription in the NR-1-infected Kasumi-3 cells. NR-1-infected Kasumi-3 cells were treated with Roscovitine at concentration of 2 μM for 48 hours and harvested for RT-qPCR analysis of IE1, UL54 and UL99 on the indicated time points along a 7-day time course. (C) Western blot analysis of IER5, CDC25B, p-CDK1 (Tyr15) and CDK1 expression in Kasumi-3 cells infected with NR-1, NR-1ΔmiR-UL148D, NR-1ΔmiR-UL148D (co-incubation with scrambled oligonucleotide) or NR-1ΔmiR-UL148D (co-incubation with miR-UL148D agomir) at 10 dpi. Left panel, representative western blot. Right panel, semi-quantitative analysis. Values are shown as the mean ± SEM (n = 3). **, P<0.01.

As previous studies have shown that CDC25B can activate CDK-1 by dephosphorylating CDK1 at Thr14 and Tyr15, which in turn inhibits HCMV IE1 transcription and promotes viral latency [13, 14, 55], we postulated that hcmv-miR-UL148-mediated upregulation of CDC25B suppresses HCMV IE1 expression by activating CDK-1. In this experiment, we replaced the Thr14 and Tyr15 residues in CDK1 with Ala14 and Phe15, respectively, to generate a constitutively activated CDK1 mutant (Fig 6A, upper panel). We then stably overexpressed wild-type CDK1 (CDK1 WT) and mutated CDK1 (T14A, Y15F) (CDK1 Mut) in Kasumi-3 cells using lentivirus vectors. The Kasumi-3 cells that were overexpressing CDK1 WT or CDK1 Mut were then infected with NR-1 or NR-1ΔmiR-UL148D. As shown in Fig 6A, overexpression of CDK1 Mut, which was constitutively activated, significantly inhibited IE1 expression in the Kasumi-3 cells infected with NR-1 or NR-1ΔmiR-UL148D, confirming that activated CDK1 can suppress IE1 expression. In contrast, overexpression of CDK1 WT did not suppress IE1 expression in the NR-1ΔmiR-UL148D-infected Kasumi-3 cells (Fig 6A). The failure of CDK1 WT to inhibit IE1 expression at a late stage of HCMV infection likely resulted from the lack of CDC25B activity, correspondingly reducing the activation of CDK1 WT. This result is in agreement with our previous findings that NR-1ΔmiR-UL148D infection results in strong inhibition of CDC25B.

We then tested whether direct inhibition of CDK1 activity in infected Kasumi-3 cells could prevent the establishment of viral latency. In this experiment, we added Roscovitine, a CDK1 inhibitor, to the culture medium of NR-1-infected Kasumi-3 cells to a final concentration of 2 μM. After 48 hours in culture, the cells were harvested, and the transcription of IE1, UL54 and UL99 was assayed at the indicated time points along the infection course (Fig 6B, upper panel). As shown in Fig 6B (lower panel), the transcription of the viral IE1, UL54 and UL99 genes increased significantly in the Roscovitine-treated cells compared to that in DMSO-treated cells. These results indicate that the activity of CDK1 in host cells plays an important role in silencing viral IE1 transcription.

We also tested whether miR-UL148D could activate CDK-1 by modulating CDC25B expression. To accomplish this, we first examined the levels of IER5, CDC25B and p-CDK1 (Tyr15) in Kasumi-3 cells infected with NR-1 or NR-1ΔmiR-UL148D at 10 dpi. As shown in Fig 6C, p-CDK1 (Tyr15) level was significantly increased and CDC25B level was significantly decreased in the Kasumi-3 cells infected with NR-1ΔmiR-UL148D compared to the mock-infected or NR-1-infected Kasumi-3 cells, suggesting that knockout of miR-UL148D indeed results in inhibition of both CDC25B and CDK1. In contrast, when the cellular miR-UL148D level in NR-1ΔmiR-UL148D-infected Kasumi-3 cells was increased by transfecting the cells with the miR-UL148D agomir, the CDC25B level increased, while the p-CDK (Tyr15) level decreased (Fig 6C), supporting that hcmv-miR-UL-148D activates CDK1 through upregulating CDC25B.

## Discussion

By screening HCMV-encoded miRNAs at various stages of experimental latent HCMV infection in CD34+ HPCs and Kasumi-3 cells, the present study demonstrated that miR-UL148D robustly accumulates during the establishment of experimental latency. Functional assays further showed that miR-UL148D possibly plays an important role in facilitating HCMV latency by modulating the IER5-CDC25B-CDK1 signaling pathway in progenitor cells.

When HCMV enters into latency, the viral IE genes are silenced and the replication cycle of the virus stops. However, recent studies have suggested that HCMV is not totally inactivated during latency; instead, various proteins, including UL138 [64], vIL-10 [65], US28 [66], and ORF94 [67], as well as a transcript that is antisense to the UL81-UL82 locus called LUNA [68], can still be detected in latently infected cells and may contribute to viral latency. For example, Humby *et al*. reported that US28 is important for latent infection of hematopoietic progenitor cells [49], and Mason *et al*. found that UL138 and LUNA can elicit immune-suppressive IL-10-producing CD4^+^ T cells to sustain latent carriage[69]. In agreement with these findings, we reported that certain HCMV miRNAs, especially miR-UL148D, robustly accumulates during the late stages of experimental latent HCMV infection. Knockout of miR-UL148D impaired the silencing of IE1 transcription in the experimental HCMV latency model. However, miR-UL148D agomir treatment effectively suppressed IE1 transcription. It is noteworthy that our mutagenic deletion of miR-UL148D also disrupted the UL150 ORF. The specific physiological function of UL150 remains unknown, although the absence or mutation of UL150 has been observed in laboratory HCMV strains [70]. It seems that the UL150 protein is not directly involved in the inhibition of IE1 because our miR-UL148D agomir treatment restored only the level of miR-UL148D and not that of UL150 in infected cells. Taken together, these results suggest that miR-UL148D possibly contributed to HCMV latency by inhibiting IE1 transcription during latent infection.

The target genes for HCMV miRNAs have not been extensively studied to date. However, it has been reported that miR-UL148D targets the chemokine RANTES and IEX-1 during HCMV infection [71, 72]. According to studies by Kim *et al*. [71] and Wang *et al*. [72], miR-UL148D suppresses the expression of RANTES or the pro-apoptotic IEX-1 in host cells as part of a viral immune evasion or anti-apoptotic strategy. However, RANTES and IEX-1 mainly function during the early stages of HCMV infection, and thus their inhibition may be irrelevant to the establishment of HCMV latency. Additionally, our data showed that miR-UL148D accumulated during late but not early stages of HCMV infection. Therefore, although RANTES and IEX-1 can be targeted by miR-UL148D, neither may play a major role in modulating HCMV latency. In the present study, using *in silico* analysis and luciferase assays, we identified that miR-UL148D directly targets IER5 by binding to two sites within the IER5 3’UTR. Mutagenesis experiments demonstrated that both sites are required for full miR-UL148D targeting and have a cooperative effect.

To assess the functional effect of miR-UL148D on IER5 downregulation, we further tested whether CDC25B expression is affected by miR-UL148D transfection (Fig 3). In the present study, we focused on the IER5-CDC25B pathway because the inhibitory effect of CDC25B on IE1 transcription through the potential modulation of CDK activity has been previously reported in host cells infected with HCMV [13, 14]. We found that miR-UL148D transfection alone lead to downregulation of IER5 expression and upregulation of CDC25B expression. Moreover, although transfection with IER5 WT plasmid alone suppressed CDC25B expression, co-transfection of IER5 WT and miR-UL148D rescued CDC25B expression in Kasumi-3 cells. In contrast, miR-UL148D could not rescue CDC25B expression when IER5 was overexpressed using an IER5 Mut plasmid in which the two miR-UL148D-binding sites in the 3’UTR of IER5 mRNA were mutated. These data demonstrate that hcmv-miR-UL148 targets IER5 to modulate the expression of CDC25B.

The identification of IER5 as a target of miR-UL148D and its role in the regulation of CDC25B are also supported by the inverse correlation between IER5 and CDC25B expression that was observed during HCMV infection in host cells. As a slow-kinetic member of the cellular immediate early response gene family and a p53 target gene, IER5 expression is significantly induced by the stimulation of DNA damage, such as through irradiation. During the early stages of NR-1 and NR-1ΔmiR-UL148D infection, IER5 expression in host cells rapidly increased, which was correlated with a decrease in CDC25B expression. This result indicates that IER5 also responds to viral DNA as a stimulus. However, during the late stages of NR-1 infection, IER5 expression was significantly downregulated and CDC25B expression was largely restored, possibly due to the high accumulation of miR-UL148D in host cells. On the contrary, during the late stages of NR-1ΔmiR-UL148D infection, IER5 expression was maintained at a high level in infected cells, while CDC25B expression was virtually undetectable. These results argue that accumulated miR-UL148D can dampen the response of IER5 to viral DNA and rescue CDC25B expression during the establishment of experimental HCMV latency.

Maintaining CDK1/2 activity has been reported as essential for successfully establishing latent HCMV infection [13, 14]. In agreement with this, our results suggest that CDC25B contributes to the establishment of HCMV latency through the activation of CDK1, which in turn inhibits the expression of the viral IE gene. As shown (Fig 5A), overexpression of IER5 in Kasumi-3 cells, which suppressed CDC25B expression, effectively prevented NR-1 virus from achieving latency. On the contrary, overexpression of CDC25B in Kasumi-3 calls restored the ability of NR-1ΔmiR-UL148D to achieve latency (Fig 5B). We directly demonstrated the inhibitory effect of CDK1 on IE1 transcription by overexpressing either a constitutively activated CDK1 mutant or CDK1 WT in host cells infected with NR-1ΔmiR-UL148D. While NR-1ΔmiR-UL148D infection resulted in sustained inhibition of CDC25B, HCMV IE1 expression was suppressed when the constitutively activated CDK1 mutant was overexpressed in host cells. Furthermore, CDK1 WT failed to suppress HCMV IE1 expression during late stages of HCMV infection. These data suggest that the IER5-CDC25B-CDK1 axis has a potential role in the silencing of HCMV IE1 transcription. Moreover, by overexpressing miR-UL148D in host cells infected with NR-1ΔmiR-UL148D and a miR-UL148D agomir, we demonstrated that miR-UL148D could activate CDK1, possibly by modulating the IER5-CDC25B pathway.

Although both CD34^+^ HPCs and Kasumi-3 cells were utilized to establish latent HCMV infections in the present study and although the results showed a similar accumulation of miR-UL148D in host cells during late-stage HCMV infection, we must acknowledge that our mechanistic study of miR-UL148D’s role in HCMV latency was mainly performed in Kasumi-3 cells. It is noteworthy that the use of Kasumi-3 cells as a viral latency model does not recapitulate all aspects of experimental HCMV latency that have been defined in primary CD34^+^ HPCs [48]. Therefore, mechanistic studies of HCMV latency in CD34^+^ HPCs or using an *in vivo* system are required for better understanding how miR-UL148D functions during latent viral infection. Previously, Meshesha *et al*. (35) reported that eight HCMV microRNAs were expressed in latently infected PBMCs by directly screening 20 HCMV miRNAs in human PBMCs. Our *in vitro* latency model using both primary CD34^+^ HPCs and Kasumi-3 cells did also suggest several HCMV miRNAs such as miR-UL112-5p, miR-UL36-5p, miR-UL22A-5p etc., is possibly latency-associated. However, miR-UL148D, suggested as a latency-associated miRNA by our *in vitro* latency model study, was not among the 20 HCMV miRNAs screened by Meshesha *et al*. (35). Therefore, the expression level and potential function of miR-UL148D during HCMV latency could be further studied using *in vivo* system in the future. Moreover, while this manuscript was under review, Lau *et al* also reported that miR-UL148D is expressed during latency and demonstrated that miR-UL148D could limit proinflammatory cytokine secretion of latently infected cells by inhibiting ACVR1B expression[73], which suggests that miR-UL148D may exert multiple function to facilitate viral latent infection. The future study could further explore the multiple role of miR-UL148D in the viral latency.

In conclusion, our study presents the first evidence that HCMV-encoded miR-UL148D robustly accumulates during the establishment of experimental HCMV latency and promotes latent HCMV infection in human progenitor cells by modulating the cellular IER5-CDC25B axis. Identifying the critical role of miR-UL148D during latent viral infection may facilitate the development of novel therapeutic approach for controlling the epidemic infection of HCMV.

## Materials and Methods

### Cells and virus

Kasumi-3 (ATCC#CRL-2725) cells were maintained in RPMI medium containing 20% fetal bovine serum (FBS) and 100 U/ml each of penicillin and streptomycin. Primary human foreskin fibroblasts (HFF-1; passages 9 to 20) (ATCC #SCRC-1041) were cultured in Dulbecco’s modified Eagle’s medium (DMEM) containing 10% FBS, 10 mM HEPES, 2 mM L-glutamine, and 100 U/ml each of penicillin and streptomycin. Bone marrow cells were obtained from waste materials used during bone marrow harvest procedures from healthy donors at Jiangsu Province People’s Hospital via a protocol approved by the Institutional Review Board of Nanjing University. All participants provided oral informed consent to study involvement. The participants received written information about the study prior to consent being obtained. As the bone marrow cells were harvested from waste materials used in a standard health examination, written consent was deemed unnecessary by the participants and the Institutional Review Board. Verbal consent was obtained by the interviewers and audited by the Institutional Review Board, and the oral informed consent process was approved by the Institutional Review Board. Primary CD34^+^ HPCs were isolated via magnetic bead-mediated cell separation (Miltenyi Biotech, Auburn, CA). Cultures of primary CD34^+^ HPCs were established as described [6, 51]. Briefly, murine AFT024 cells were plated in 6-well plates coated with 0.1% gelatin (Stem Cell Technologies, Vancouver, CA) and irradiated. CD34^+^ cells were cultured in RPMI medium supplemented with 20% FBS, 100 μM 2-mercaptoethanol, and 10 ng/ml each of stem cell factor, Flt-3 ligand, and IL-7 (R&D Systems, Minneapolis, MN) and plated in Transwell plates with 0.4 μM microporous filters (Costar, Cambridge, MA) above the irradiated AFT024 cells. The cells were cultured in an incubator with 5% CO_2_ at 37°C. Murine AFT024 (ATCC#SCRC-1007) cells were maintained in DMEM supplemented with 10% FBS and 50 μM 2-mercaptoethanol at 32°C.

The bacterial artificial chromosome (BAC)-derived clinical strain NR-1 was used in this study. For infections, NR-1 expressing enhanced green fluorescent protein (eGFP) was used. NR-1ΔmiR-UL148D was generated using a bacterial recombineering method as previously described [11]. In brief, the UL150 region that encoded miR-UL148D was specifically deleted in NR-1 by inserting a Kana cassette. First, Kana cassettes were PCR-amplified using the following primers: forward, 5’-GCCGGTCTCGGAGACCGTGGACGAAAAAGAGAACGCAGCAGCTATCGCTGGC GGAGCGCTCTCGCGTTGCATTTTTGTTC-3’; reverse, 5’-TTCCAGCCCTGCCACGCCCAACG CGGCACTTCCAACAGAGCCACATCCCAGAGGGTATTGGCCCCAATGGGGTCTCGGTG-3’. The underlined nucleotides correspond to the Kana gene. The sequences that are not underlined represent 54 nucleotides upstream and downstream of pre-miR-UL148D. The amplified DNA fragments were introduced into *E*. *coli* EL350 cells containing a wild-type NR-1 bacterial artificial chromosome (BAC) for recombination by electroporation (Bio-Rad, Hercules, CA). The miR-UL148D-deleted NR-1 BAC construct containing the Kana cassette was selected on Luria Broth (LB) plates containing kanamycin. The NR-1ΔmiR-UL148D BAC vector was then introduced into human foreskin fibroblast cells (HFF cells) by electroporation (Bio-Rad, Hercules, CA). Virus particles were harvested from the cells when they showed a complete cytopathic effect, and virus titers were measured with PFU assays of fibroblasts. HCMV was propagated in primary human foreskin fibroblast cells in DMEM supplemented with 10% FBS and 100 U/ml each of penicillin and streptomycin. Virus stocks were stored in DMEM containing 10% FBS and 1.5% bovine serum albumin (BSA) at -80°C.

### Viral infection and reactivation

Kasumi-3 cells and primary CD34^+^ HPCs were infected with NR-1 or NR-1ΔmiR-UL148D at a multiplicity of 5 PFU/cell. UV-inactivated virus was used for mock infection. One hour before infection, the cell culture medium was replaced with a serum-free medium. The infected cells were incubated for 4 h at 37°C and 5% CO_2_ and then washed three times with PBS to remove cell-free virus. The infected cells were then harvested at the indicated time points post-infection. The culture medium was changed for all cells every day. For virus reactivation in infected Kasumi-3 cells, 20 nM 12-O-tetradecanoylphorbol acetate (TPA) (Sigma-Aldrich, St Louis, MO) was added to the latently infected cell culture for 48 hours to induce viral lytic gene reactivation. For virus reactivation in infected primary CD34^+^ HPCs, the latently infected cells were transferred to reactivation medium [51] containing a modification of Eagle’s medium supplemented with 20% FBS, 1 μM hydrocortisone, 0.02 mM folic acid, 0.2 mM *i*-inositol, 0.1 mM 2-mercaptoethanol, 2 mM L-glutamine, 100 U/ml each of penicillin and streptomycin, and 15 ng/ml of each of the following cytokines: IL-6, granulocyte colony-stimulating factor (G-CSF), granulocyte-macrophage colony-stimulating factor (GM-CSF), and IL-3 (R&D Systems). For the co-culture experiments, latently infected or mock-infected Kasumi-3 cells and HPCs were cultured under conditions favoring lytic reactivation (described above) or control conditions for 6 days. Cells were then washed with PBS and co-cultured with HFFs for 2 days. Then, the Kasumi-3 cells were removed from the co-cultures, and the HFFs were washed with PBS and cultured for an additional 5 days for fluorescence microscopy analysis of GFP-positive plaques.

### Incubation with miR-UL148D agomir during HCMV infection

A miR-UL148D agomir was purchased from RiboBio Co. (Guangzhou, China). The agomir is composed of a synthetic miR-UL148D duplex chemically modified for greater stability and shown to produce gain of function *in vivo*. The miR-UL148D agomir was added to cell culture medium at a final concentration of 1 mM. Kasumi-3 cells were incubated with the agomir at 37°C for 24 h prior to NR-1ΔmiR-UL148D infection, and the culture medium was changed every day with the addition of fresh miR-UL148D agomir.

### Plasmid construction and transfection

To generate luciferase reporter plasmids, the full-length IER5 3’UTR with or without mutated binding sites was chemically synthesized and cloned into a pMIR reporter plasmid (Ambion, Austin, TX). Successful insertion was confirmed by sequencing. For luciferase reporter assays, 0.2 μg of a firefly luciferase reporter plasmid, 0.1 μg of a β-galactosidase expression vector (Ambion, Austin, TX) and equal amounts (20 pmol) of HCMV miR-UL148D mimics or scrambled negative control RNA were transfected into 239T cells in 24-well plates using Lipofectamine 3000 (Life Technology, Carlsbad, CA). The β-galactosidase vector was used as a transfection control. At 24 h post-transfection, the transfected cells were analyzed using a luciferase assay kit according to the manufacturer’s instructions (Promega, Madison, WI). To generate IER5 expression plasmids, full-length IER5 cDNA sequences with or without mutated binding sites were chemically synthesized and cloned into a pcDNA3.1(+) vector. Successful insertion was confirmed by sequencing. Then, the IER5-expressing plasmid (3 μg) and the β-galactosidase expression vector (1 μg) were co-transfected into Kasumi-3 cells cultured in 6-well plates with 100 pmol of miR-UL148D mimics or scrambled negative control RNA using Lipofectamine 3000. Cells transfected with the β-galactosidase vector alone served as a transfection control. At 72 h post-transfection, the cells were harvested for western blotting.

### Infection with lentivirus

Lentiviruses encoding IER5, CDC25B, CDK1 WT, and CDK AF mutant genes were generated and confirmed by the GenePharma Company (Shanghai, China). An empty-backbone lentivirus was used as a control. Cells were incubated with the lentiviruses at a multiplicity of infection (MOI) of 5:1 along with 8 μg/ml Polybrene for 48 h before further treatment. The selection marker was GFP. The infected cells were gated for GFP expression during flow cytometry analysis.

### Western blotting

Cells from three independent experiments were harvested at the indicated time points after transfection or infection. Western blotting was performed using the following primary antibodies: goat polyclonal anti-IER5 antibody, rabbit polyclonal anti-CDC25B antibody, mouse monoclonal anti-GAPDH antibody (all from Santa Cruz), and rabbit monoclonal anti-CDK1 and anti-p-CDK1 (Tyr15) antibodies (Cell Signaling Technology, Danvers, MA).

### Analysis of HCMV miRNA

In total, 20,000 infected cells were harvested for the isolation of total RNA and DNA at each indicted time point. Fourteen HCMV miRNAs were assayed using a human cytomegalovirus miRNA probe kit according to the manufacturer’s protocol (ThermoFisher Scientific, Waltham, MA). Equal amounts of cDNA were analyzed by qPCR in triplicate using an Applied Biosystems 7500 Real-time PCR machine. The amount of each HCMV miRNA was then calculated with a standard curve. Viral genomic DNA was first quantified by qPCR, and then the absolute viral genomes copy number was calculated with a standard curve. The levels of the HCMV miRNAs were normalized to the viral genome copies to obtain the relative levels of the miRNAs.

### Analysis of viral RNA transcription and DNA copy number

The infected cells were collected by low-speed centrifugation and washed with PBS three times. Cell-associated viral DNA was isolated as described previously [50]. Viral genome copy number was evaluated by qPCR (normalized to the cellular gene GAPDH) and semi-quantitative PCR using the IE1 primer. For the qPCR experiment, all samples were analyzed in triplicate using the SYBR Green Probe and an Applied Biosystems 7500 Real-time PCR machine. All samples were analyzed by PCR for 28 cycles and then submitted to agarose electrophoresis. The absolute HCMV genome copy number was then determined according to a standard curve. Briefly, the IE1 ORF was synthesized and diluted into samples containing a series of different copy numbers. The samples were used as templates for analysis in triplicate with the SYBR Green probe on the Applied Biosystems 7500 Real-time PCR machine to generate a concentration standard curve. Then, the Ct value for cell-associated viral DNA was determined through equal loading of total infected cellular DNA (0.5 μg). Through the standard curve, the copy numbers of cell-associated viral DNA were determined. Intracellular viral RNA was assayed as described previously [50]. In brief, RNA was isolated using Trizol Reagent (Life Technology, Carlsbad, CA) according to the manufacturer’s instructions. The RNA samples were treated with DNase using a DNA-free kit (Ambion, Austin, TX) according to the manufacturer’s instructions. The concentration of RNA was then determined, and 0.5 μg was used for a reverse transcriptase (RT) reaction. IE1 cDNA was synthesized using a Reverse Transcription kit with random hexamers according to the manufacturer’s protocol (Applied Biosystems). Equal amounts of cDNA were analyzed by quantitative PCR (qPCR) in triplicate using an Applied Biosystems 7500 Real-time PCR machine. RNA was normalized to cellular GAPDH. cDNA quantity was also analyzed by semi-qPCR. For the semi-qPCR, all samples were analyzed using a 28-cycle reaction and subsequent agarose electrophoresis. The primer sets used to amplify DNA and cDNA of IE1, UL138, UL54, UL99 are as follows: IE1, forward 5’-GCCTTCCCTAAGACCACCAAT-3’ and reverse 5’-ATTTTCTGGGCATAAGCCATAATC-3’; UL138, forward 5’-TGCGCATGTTTCTGAGCTAC-3’ and reverse 5’-ACGGGTTTCAACAGATC GAC-3’; UL54, forward 5’-CCCTCGGCTTCTCACAACAAT-3’, and reverse 5’-CGAGGTAGT CTTGGCCATGCAT-3’; UL99, forward 5’-GTGTCCCATTCCCGACTCG-3’ and reverse 5’-TTC ACAACGTCCACCCACC-3’. The primers sets used to amplify DNA and cDNA of GAPDH are as follows: forward 5’-TCAGAAAAAGGGCCCTGACAACT-3’, reverse 5’-TCCCCTCTTCAAG GGGTCTACA-3’; forward 5’-AGGCTAGGGACGGCCT-3’, reverse 5’-GCCATGGGTGGAATCAT ATTG-3’.

### Immunofluorescence analysis

Infected cells were harvested after the indicated treatments by centrifugation at 300×g for 5 minutes at 4°C and processed for immunofluorescence microscopy. Briefly, the harvested cells were washed using cold phosphate-buffered saline (PBS) and fixed with 4% paraformaldehyde for 10 minutes at room temperature. Then, the cells were permeabilized with PBS containing 0.2% (v/v) Triton X-100 for 5 minutes at room temperature. After being blocked with bovine serum albumin (BSA) for 1 hour at room temperature, the cells were incubated with IE1 antibody (clone 1B12) [74] (1:100) in PBS containing 10 mg/ml BSA overnight at 4°C. The cells were then washed with PBS and incubated with Alexa Flour 594 (goat anti-rabbit IgG, 1:2000) for 1 h at room temperature. The cells were then washed using PBS, mounted and analyzed for GFP (green), IE1 (red), and DAPI (blue) using an inverted confocal laser microscope (Nikon).

### Enzyme-linked immunosorbent assay (ELISA)

Anti-HCMV IgG and IgM antibodies in plasma were detected by ELISA using an HCMV IgG/IgM kit (DIA PRO Diagnostic Bioprobes, Milano, Italy) according to the manufacturer’s instructions. For the IgG-ELISA, an ELISA value of <0.5 IU/ml was considered negative and a value of >0.5 IU/ml was considered positive, indicating prior exposure to HCMV. For the IgM-ELISA, the test results were calculated using the optical density (OD) value at 450 nm, and the cut-off value for positivity was OD > 1.2, indicating acute infection with HCMV.

### Statistical analysis

Data derived from at least three independent experiments were presented as the mean ± SEM. Normal distributed variables were compared using Student’s *t*-test. The reported *P* values are 2-sided. *P* < 0.05 was considered statistically significant.

## Supporting Information

S1 Fig Infection efficiency of different strains of HCMV and viability of infected cells detected by double fluorescence labeling. Three strains of HCMV virus, NR-1 (wild type and miR-UL148D mutated), Towne and AD169 were used to infected Kasumi-3 and CD34^+^ HPCs (MOI of 5). At 48 hours post-infection, cells were harvested to assay the GFP and Annexin V levels by flow cytometry.
(TIF)

S2 Fig Isolation of primary CD34^+^ HPCs from the bone marrow of five HCMV-IgM and HCMV-DNA negative donors by anti-CD34 magnetic cell separation kit. (A) Human cytomegalovirus infection status of five bone marrow donors. Anti-HCMV IgG and IgM antibodies in plasma was detected by ELISA using an HCMV IgG/IgM kit and HCMV DNA was assayed by qPCR. (B) The percentage of CD34^+^ HPCs in sorted HPCs and Mock- or NR-1-infected HPCs on 10 dpi.
(TIF)

S3 Fig Infection of NR-1ΔmiR-UL148D fails to produce miR-UL148D in host cells. (A) Multiple-step growth (multiplicity of infection, MOI = 0.05) of NR-1 and NR-1ΔmiR-UL148D in HFF. At different time points post-infection, both cells and culture medium samples were harvested and sonicated for miR-UL148D and viral titer assay. The viral titers were determined by plaque assays on HFF. (B) The miR-UL148D level in HFF cells infected with NR-1 or NR-1ΔmiR-UL148D on 4 day post-infection. The total RNA was isolated and assayed with miR-UL148D probe.
(TIF)

S4 Fig Representative results of infection efficiency by various lentivirus constructs. (A) Cells were incubated with respective virus at a MOI of 5 along with 8μg/ml Polybrene for 48 hours before the following treatment. The selection marker was GFP. The infected cells were gated by GFP expression via flow cytometry analysis.
(TIF)
