# Supplementary figures and images for: Human Cytomegalovirus miR-UL148D Facilitates Latent Viral Infection by Targeting Host Cell Immediate Early Response Gene 5
Source: PLoS Pathog. 2016 Nov 8;12(11):e1006007. doi: 10.1371/journal.ppat.1006007 (PMC5100954; doi:10.1371/journal.ppat.1006007)

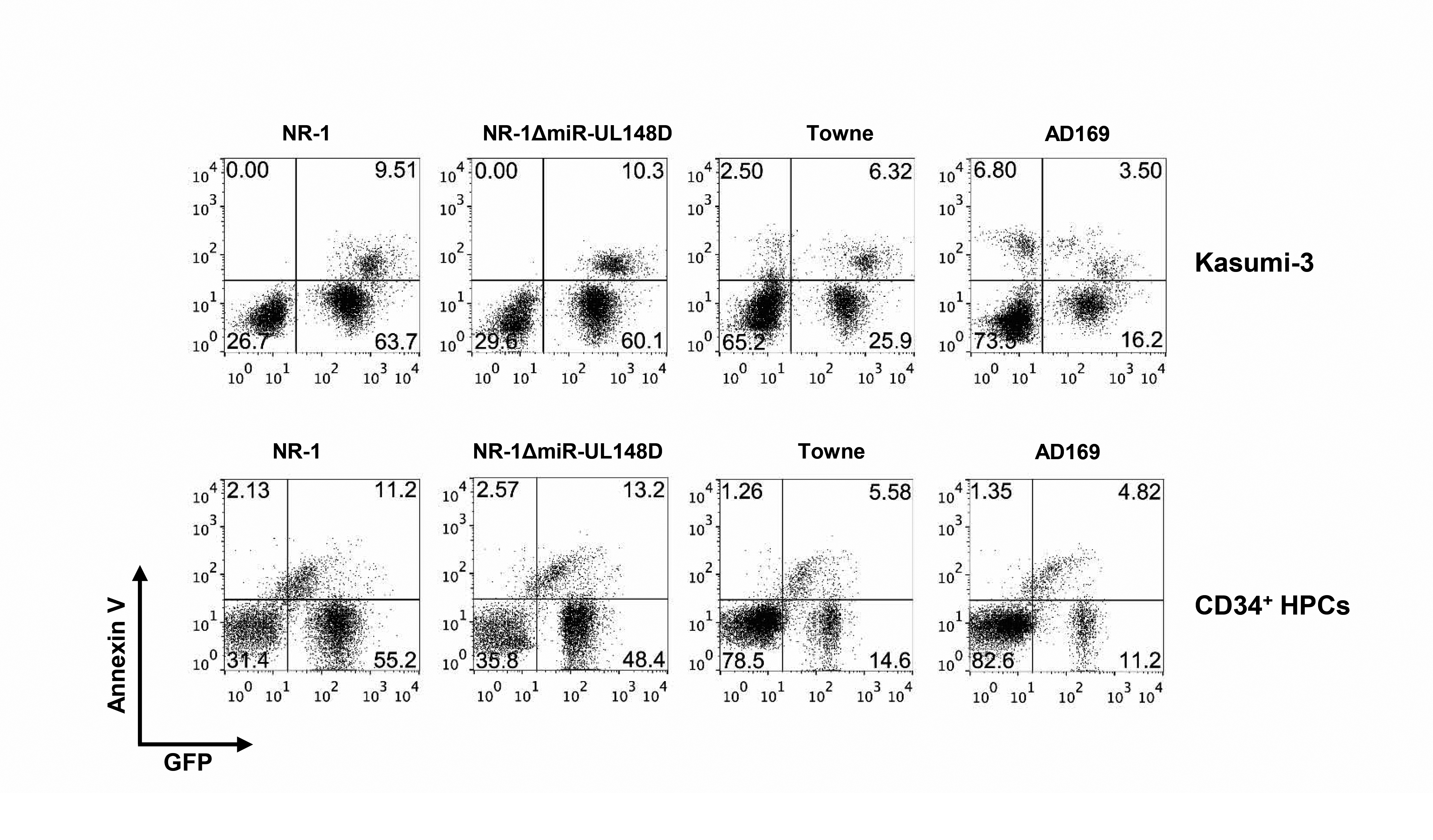

Supplement: S1 Fig — Three strains of HCMV virus, NR-1 (wild type and miR-UL148D mutated), Towne and AD169 were used to infected Kasumi-3 and CD34+ HPCs (MOI of 5). At 48 hours post-infection, cells were harvested to assay the GFP and Annexin V levels by flow cytometry. (TIF) [file ppat.1006007.s001.tif]

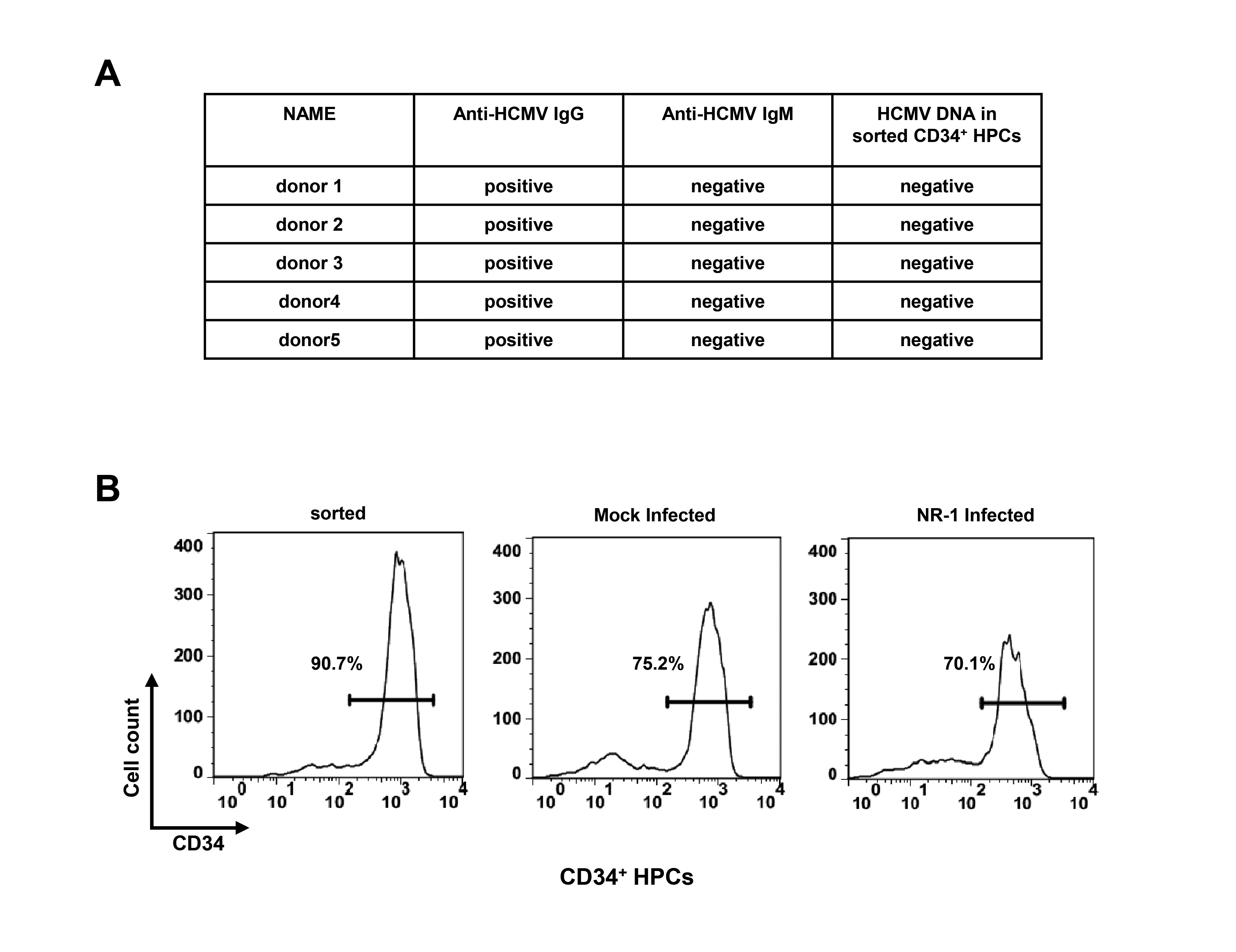

Supplement: S2 Fig — (A) Human cytomegalovirus infection status of five bone marrow donors. Anti-HCMV IgG and IgM antibodies in plasma was detected by ELISA using an HCMV IgG/IgM kit and HCMV DNA was assayed by qPCR. (B) The percentage of CD34+ HPCs in sorted HPCs and Mock- or NR-1-infected HPCs on 10 dpi. (TIF) [file ppat.1006007.s002.tif]

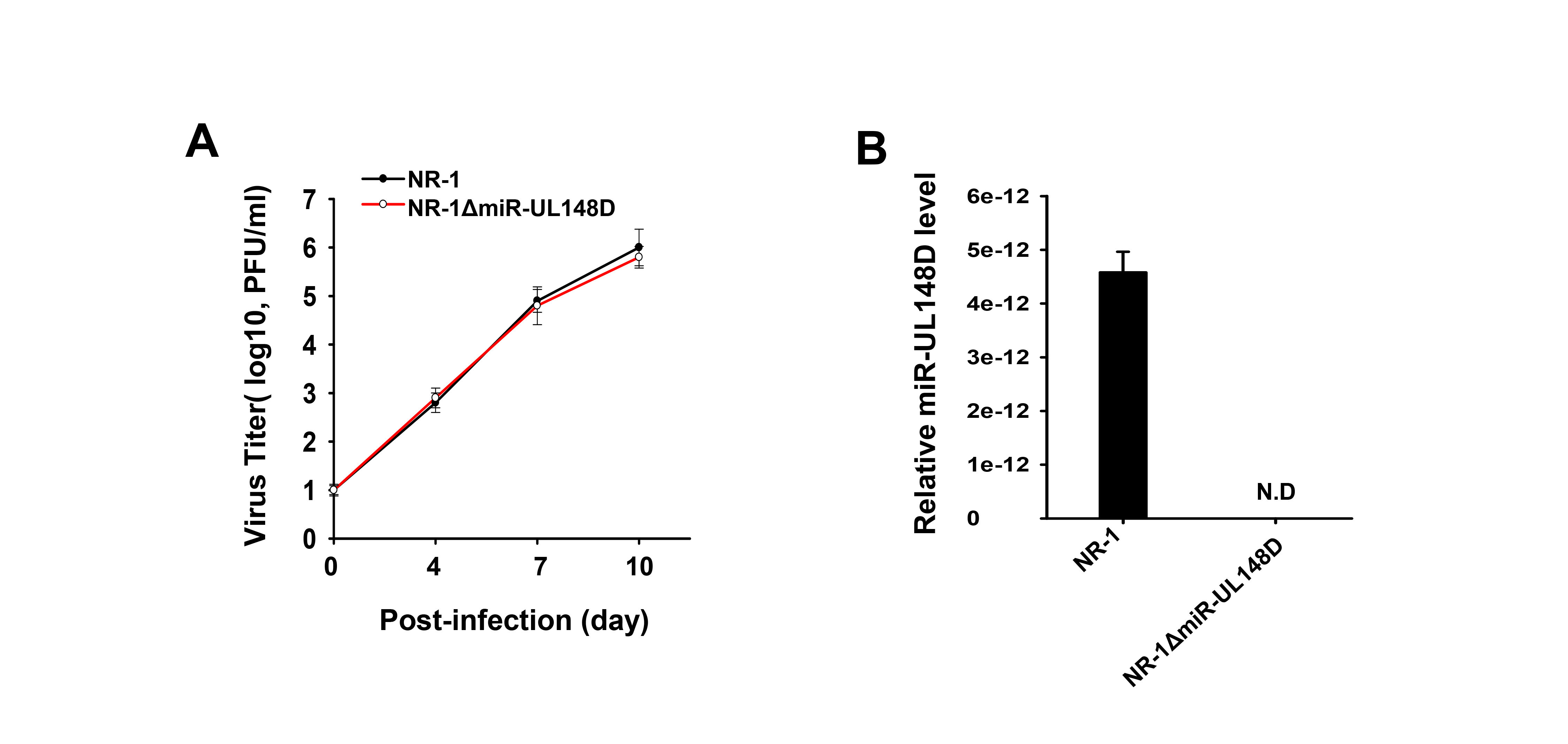

Supplement: S3 Fig — (A) Multiple-step growth (multiplicity of infection, MOI = 0.05) of NR-1 and NR-1ΔmiR-UL148D in HFF. At different time points post-infection, both cells and culture medium samples were harvested and sonicated for miR-UL148D and viral titer assay. The viral titers were determined by plaque assays on HFF. (B) The miR-UL148D level in HFF cells infected with NR-1 or NR-1ΔmiR-UL148D on 4 day post-infection. The total RNA was isolated and assayed with miR-UL148D probe. (TIF) [file ppat.1006007.s003.tif]

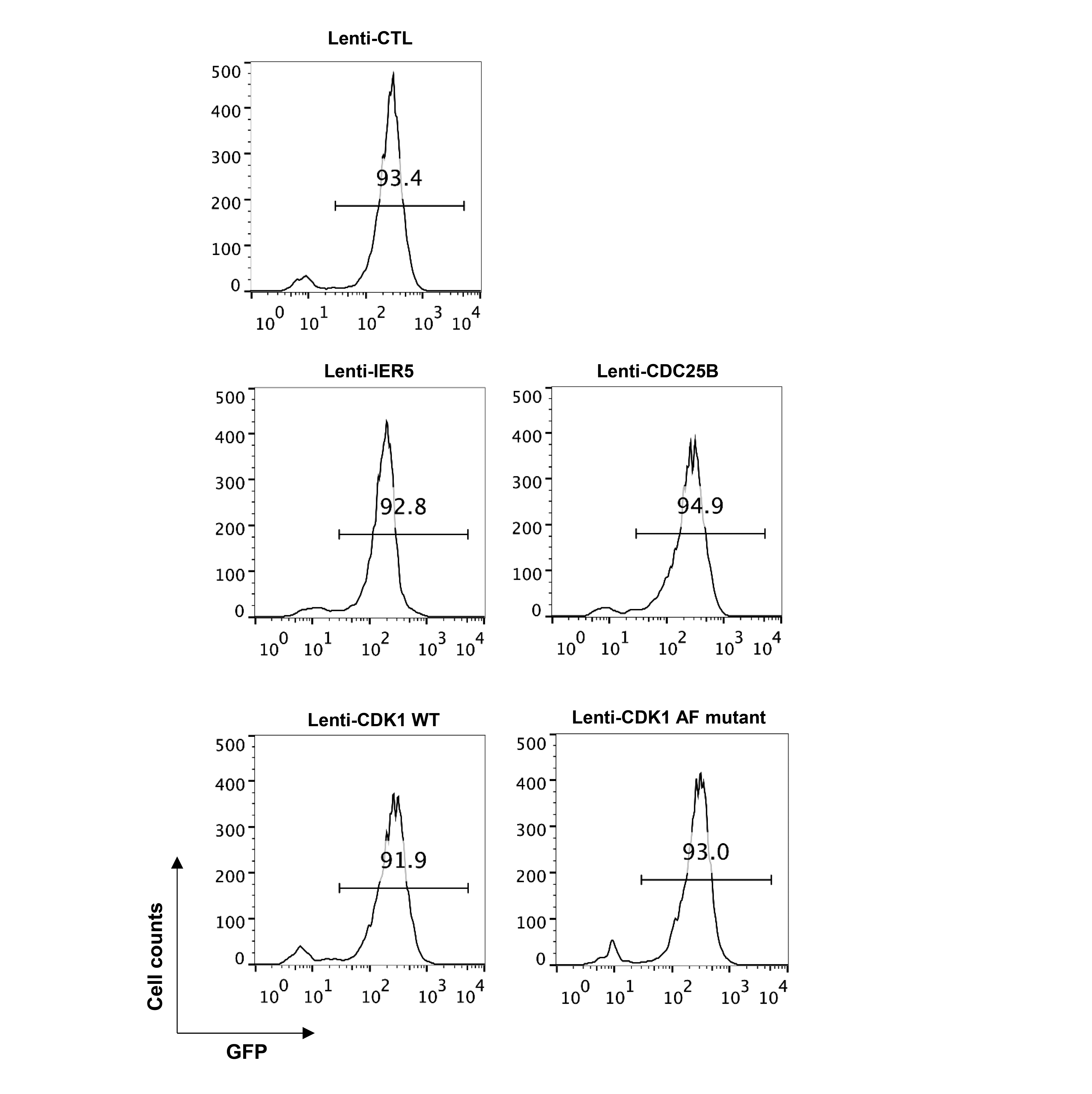

Supplement: S4 Fig — (A) Cells were incubated with respective virus at a MOI of 5 along with 8μg/ml Polybrene for 48 hours before the following treatment. The selection marker was GFP. The infected cells were gated by GFP expression via flow cytometry analysis. (TIF) [file ppat.1006007.s004.tif]
